# Supplementary material for: Cancer Worry Distribution and Willingness to Undergo Colonoscopy at Three Levels of Hypothetical Cancer Risk—A Population-Based Survey in Sweden
Source: Cancers (Basel). 2022 Feb 12;14(4):918. doi: 10.3390/cancers14040918 (PMC8870195; doi:10.3390/cancers14040918)
Supplement: Supplementary file 1 [file cancers-14-00918-s001.zip › cancers-1575752-supplementary file1.pdf]

Supplementary Materials: Cancer Worry Distribution and Willingness to Undergo Colonoscopy at Three Levels of Hypothetical Cancer Risk—A Population-Based Survey in Sweden

Carolina Hawranek, Johan Maxon, Andreas Andersson, Bethany Van Guelpen, Senada Hajdarevic, Barbro Numan Hellquist and Anna Rosén

Tools used in the survey

Table S1. The Cancer Worry Scale (translated to English)

The first 6 items (referred to as 6-item CWS) are presented in the main article. The full 8-item CWS data are presented in this appendix.

Item	Likert scale response options (point)			
1. How often have you thought about your risk of developing cancer (again)?	Seldom or never (1)	Sometimes (2)	Often (3)	Almost all the time (4)
2. Have these thoughts affected your mood?	Not at all (1)	A little (2)	Somewhat (3)	A lot (4)
3. Have these thoughts interfered with your ability to do daily activities?	Not at all (1)	A little (2)	Somewhat (3)	A lot (4)
4. How worried are you about the risk of developing cancer (again) one day?	Not at all worried (1)	Not very worried (2)	Pretty worried (3)	Very worried (4)
5. How often do you worry about developing cancer (again)?	Seldom or never (1)	Sometimes (2)	Often (3)	Almost all the time (4)
6. How much of a problem is this worry?	Very small (1)	Pretty small (2)	Pretty large (3)	Very large (4)
7. How often do you worry about the risk of family members developing cancer?	Seldom or never (1)	Sometimes (2)	Often (3)	Almost all the time (4)
8. How worried are you about the risk that you will ever need surgery for cancer (again)?	Not at all worried (1)	Not very worried (2)	Pretty worried (3)	Very worried (4)

Table S2. The original cancer worry scale items (in Swedish)

Fråga	Svarsalternativ (poäng)			
1. Hur ofta har du tänkt på risken att utveckla cancer (eller att utveckla cancer igen)?	Sällan eller aldrig (1)	Ibland (2)	Ofta (3)	Nästan hela tiden (4)
2. Har dessa tankar påverkat ditt humör?	Inte alls (1)	Lite (2)	En del (3)	Mycket (4)
3. Har dessa tankar påverkat din förmåga att utföra dagliga sysslor?	Inte alls (1)	Lite (2)	En del (3)	Mycket (4)
4. Hur orolig är du över risken att någon gång utveckla cancer (eller att utveckla cancer igen)?	Inte alls orolig (1)	Inte särskilt orolig (2)	Ganska orolig (3)	Mycket orolig (4)
5. Hur ofta oroar du dig över att utveckla cancer (eller att utveckla cancer igen)?	Sällan eller aldrig (1)	Ibland (2)	Ofta (3)	Nästan hela tiden (4)
6. Hur stort problem är denna oro?	Mycket litet (1)	Ganska litet (2)	Ganska stort (3)	Mycket stort (4)
7. Hur ofta oroar du dig för risken att dina familjemedlemmar kommer att utveckla cancer?	Sällan eller aldrig (1)	Ibland (2)	Ofta (3)	Nästan hela tiden (4)

8. Hur orolig är du över möjligheten att du någon gång behöver opereras för cancer (eller behöver opereras för cancer igen)?	Inte alls orolig (1)	Inte särskilt orolig (2)	Ganska orolig (3)	Mycket orolig (4)

Table S3. The CRC screening-scenarios (translated to English)

Quote from introductory paragraph: "During a bowel examination one can detect, and remove, precursors of colon cancer early. The chance of being cured increases if this form of cancer is detected early. The examination involves the insertion of a soft flexible tube with a camera in the rectum, which can be perceived as unpleasant."

Question	Likert scale response options			
1. Would you like to undergo a bowel examination at one time if you had a normal risk (about 5 percent) of getting colon cancer?	No, absolutely not	No, I don't think so	Yes, I think so	Yes, absolutely
2. Would you like to undergo a bowel examination every five years if you had a doubled risk (about 10 percent) of getting colon cancer?	No, absolutely not	No, I don't think so	Yes, I think so	Yes, absolutely
3. Would you like to undergo a bowel examination every second year if you had a greatly increased (approximately 70 percent) risk of getting colon cancer?	No, absolutely not	No, I don't think so	Yes, I think so	Yes, absolutely

<u>*Subgroup without personal cancer history (n=853)*</u>

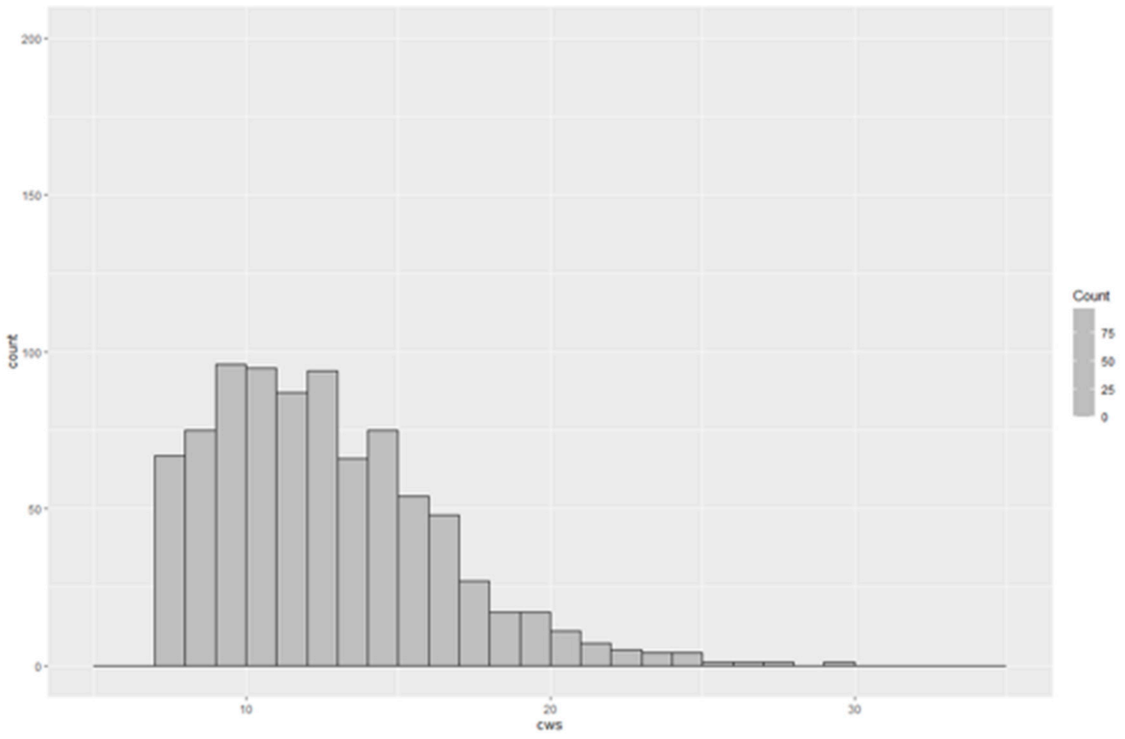

Figure S1. Histogram of 8-item CWS-score in respondents without personal cancer history

Table S4. 8-item CWS scores in participants without personal cancer history

		Women (n=403)					Men (n=450)				
	Subgroup	Mean (stand.dev.)	P-value t-test/ ANOVA	CWS score 8-13 N (%)†	CWS score 14-32 N (%)†	P-value Chi2	Mean (stand.dev.)	P-value t-test/ ANOVA	CWS score 8-13 N (%)†	CWS score 14-32 N (%)†	P-value Chi2
**Total**	-	13.74 (3.95)		213 (53)	190 (47)		12.51 (3.36)		301 (67)	149 (33)	
**Age**	18-29	13.42 (3.81)		36 (61)	23 (39)		12.23 (3.54)		47 (73)	17 (27)	
	30-39	14.64 (4.75)		25 (45)	31 (55)		12.67 (3.75)		52 (66)	27 (34)	
	40-49	13.56 (4.05)		42 (59)	29 (41)		12.59 (3.48)		52 (63)	30 (37)	
	50-59	13.39 (3.61)		43 (57)	32 (43)		13.32 (3.37)		38 (58)	28 (42)	
	60-69	13.79 (3.87)		37 (46)	43 (54)		12.26 (2.86)		68 (71)	28 (29)	
	70-74	13.77 (3.70)		30 (48)	32 (52)		12.05 (3.12)		44 (70)	19 (30)	
			P =.85			*P* =.24		*P* =.61			*P* =.39
**Education** ‡	Lower	13.88 (3.98)		78 (54)	67 (46)		12.54 (3.40)		129 (65)	70 (35)	
	Middle	14.18 (4.24)		58 (45)	70 (55)		12.63 (3.24)		92 (64)	51 (36)	
	Higher	13.17 (3.59)		75 (59)	52 (41)		12.31 (3.47)		79 (74)	28 26)	
			P =.11			*P* =.09		*P* =.75			*P* =.21

**Country of birth**[§]	Sweden	13.78 (3.96)		193 (52)	178 (48)		12.39 (3.23)		281 (68)	133 (32)	
	Other	13.04 (3.66)		17 (63)	10 (37)		13.85 (4.60)		19 (58)	14 (32)	
			P =.32			*P* =.37		*P* =.08			*P* =.31
**Children**[¶]	Yes	13.86 (3.80)		134 (51)	131 (49)		12.63 (3.30)		180 (65)	95 (35)	
	No	13.45 (4.21)		77 (57)	57 (43)		12.30 (3.42)		121 (70)	53 (30)	
			P =.34			*P* =.23		*P* =.32			*P* =.43

[†]Numbers may not sum to 100 due to rounding. [‡]Education categorized into Lower (high school or less), Middle (up to 2 years at post secondary level) or Higher (over 2 years at post secondary level). NA (n=4) not included. [§]Country of birth with response options; Sweden, Europe or Outside Europe clustered into Sweden and Other. NA (n=8) not included.
[¶] Respondents' answers to the question; "Do you have children?". NA (n=5) not included.

Population-based sample

Table S5. Characteristics of respondents and non-respondents

		**Non-respondents** (including non-completes[†])	**Respondents**	**Chi^2 test** respondents vs non-respondents	**Respondents** with cancer history[‡]	**Respondents** without cancer history[‡]	**Chi^2 test** respondents with vs without cancer history
	Subgroup	N (%)[§]	N (%)[§]		N (%)[§]	N (%)[§]	
**Total**	-	857	943		82	853	
**Gender**	Men	415 (48)	495 (52)	*P* =.094	41 (50)	450 (53)	*P* =.72
	Women	442 (52)	448 (48)		41 (50)	403 (47)	
	NA	0	0		0	0	
**Age**	18-29	263 (31)	123 (13)	*P* <.001***	0 (0)	123 (14)	*P* <.001***
	30-39	193 (23)	141 (15)		4 (5)	135 (16)	
	40-49	164 (19)	160 (17)		7 (9)	153 (18)	
	50-59	87 (10)	155 (16)		12 (15)	141 (17)	
	60-69	99 (12)	205 (22)		25 (30)	176 (21)	
	70-74	51 (6)	159 (17)		34 (41)	125 (15)	
	NA	0	0		0	0	
**Education**[¶]	Lower	366 (43)	378 (40)	*P* =.043*	30 (37)	344 (40)	*P* =.83
	Middle	237 (28)	301 (32)		27 (33)	271 (32)	
	Higher	190 (22)	259 (27)		24 (29)	234 (27)	
	NA	64 (7)	5 (1)		1 (1)	4 (0)	
**Country of birth**[††]	Sweden	671 (78)	872 (92)	*P* =.018*	80 (98)	785 (92)	*P* =.17
	Other	75 (9)	63 (7)		2 (2)	60 (7)	
	NA	111 (13)	8 (1)		0 (0)	8 (1)	
**Children**[‡‡]	Yes	403 (47)	318 (34)	*P* <.001***	73 (89)	540 (63)	*P* <.001***
	No	376 (44)	620 (66)		9 (11)	308 (36)	
	NA	78 (9)	5 (1)		0 (0)	5 (1)	

[†]47 responded to some but not all 8 CWS items (non-completers). These non-completers may be a different population than the non-respondents but including them among non-respondents did not change the differences between respondents and non-respondents significantly. [‡]8 individuals did not reply to the question about personal cancer history (yes/no).[§] Numbers may not sum to 100 due to rounding. [¶]Education clustered into Lower

(high school or less), Middle (up to 2 years at post secondary level) or Higher (over 2 years at post secondary level). ††Country of birth with response options; Sweden, Europe or Outside Europe clustered into Sweden and Other. ‡‡Respondents' answers to the question; "Do you have children?".

Table S6. Characteristics of the respondents compared with the Swedish population aged 18-74 during 2018

	Subgroup	Respondents	Swedish population†	Chi^2 test
		N (%)‡	N (%)‡	
**Total**	-	943	7 152 054	
**Gender**	Men	495 (52)	3 633 651(51)	*P* =.29
	Women	448 (48)	3 518 403 (49)	
	NA	0	0	
**Age**	18-29	123 (13)	1 562 778 (22)	*P* <.001**
	30-39	141 (15)	1 330 260 (19)	
	40-49	160 (17)	1 294 175 (18)	
	50-59	155 (16)	1 286 816 (18)	
	60-69	205 (22)	1 114 377 (16)	
	70-74	159 (17)	563 648 (8)	
	NA	0	0	
**Education§**	Lower	378 (40)	4 219 613 (59)	*P* <.001**
	Middle	301 (32)	1 072 193 (15)	
	Higher	259 (28)	1 680 357 (23)	
	NA	5 (1)	179 891 (3)	
**Country of birth¶**	Sweden	872 (92)	5 537 132 (77)	*P* <.001**
	Other	63 (7)	1 614 922 (23)	
	NA	8 (1)	0	
**Children††**	Yes	620 (66)	4 577 315 (64)	*P* =.19
	No	318 (34)	2 574 739 (36)	
	NA	5 (1)	0	

†Swedish population data on number of individuals aged 18-74 years in 2018 retrieved from officially available reports by Statistics Sweden (SCB). ‡ Numbers may not sum to 100 due to rounding. §Education clustered into Lower (high school or less), Middle (up to 2 years at post secondary level) or Higher (over 2 years at post secondary level). NA (n=4) not included. ¶Country of birth with response options; Sweden, Europe or Outside Europe clustered into Sweden and Other. ††Respondents' answers to the question; "Do you have children?".

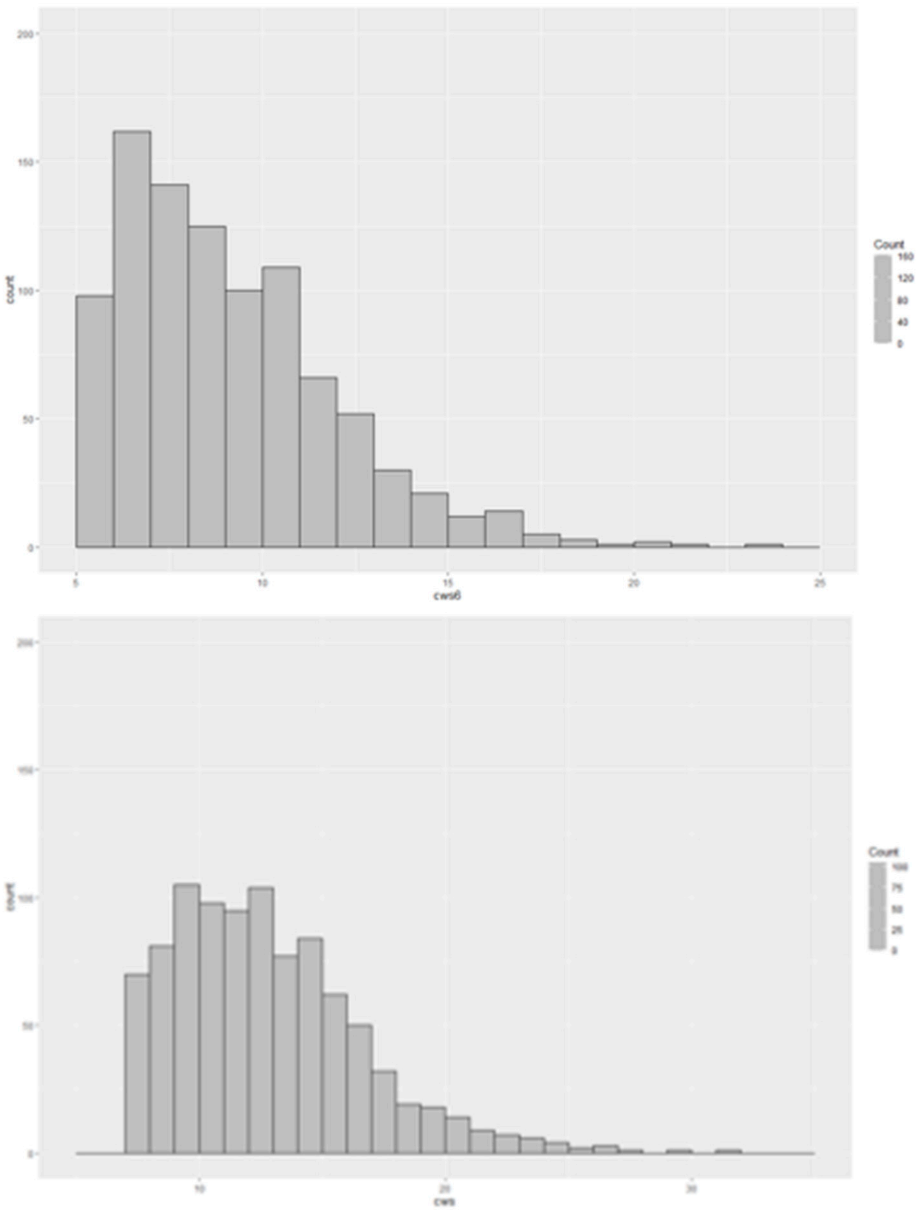

Figure S2. Histogram of the 6-item (mean 9.61, median 9) and 8-item CWS-scores (mean 13.26, median 13) in the population-based sample

Table S7. Scores of the 6-item CWS in the population-based sample

	Subgroup	6-item CWS score Median (min-max)	Mean (SD)	P-value t-test/ ANOVA	6-item CWS-score interval Low 6-11 N (%)†	High 12-24 N (%)†	P-value Chi2
**Total**	-	9 (6-24)	9.61 (2.87)		735 (78)	208 (22)	
**Gender**	Women	10 (6-22)	10.10 (3.09)	*P*<.001**	329 (73)	119 (27)	*P*<.002**
	Men	9 (6-24)	9.17 (2.59)		406 (82)	89 (18)	
**Age**	18-29	9 (6-19)	9.27 (2.78)	*P*=.93	98 (80)	25 (20)	*P*=.51
	30-39	9 (6-22)	9.79 (3.26)		104 (74)	37 (26)	
	40-49	9 (6-24)	9.66 (3.14)		123 (77)	37 (23)	
	50-59	9 (6-20)	9.83 (2.84)		117 (75)	38 (25)	
	60-69	9 (6-21)	9.61 (2.68)		162 (79)	43 (21)	
	70-74	9 (6-18)	9.44 (2.56)		131 (82)	28 (18)	
**Education**	Lower	9 (6-22)	9.62 (2.83)	*P*=.32	290 (77)	88 (23)	*P*=.63
	Middle	9 (6-24)	9.76 (2.99)		234 (78)	67 (22)	
	Higher	9 (6-17)	9.39 (2.78)		207 (80)	52 (20)	
**Country of birth**	Sweden	9 (6-24)	9.58 (2.83)	*P*=.47	682 (78)	190 (22)	*P*=.61
	Other	9 (6-21)	9.91 (3.45)		47 (75)	16 (25)	
**Children**	Yes	9 (6-24)	9.74 (2.85)	*P*=.03*	476 (77)	144 (23)	*P*=.22
	No	9 (6-22)	9.32 (2.89)		256 (80)	62 (20)	
**Cancer history††**	Yes	11 (6-24)	11.00 (3.63)	*P*<.001**	56 (68)	26 (32)	*P* =.04*
	No	9 (6-22)	9.46 (2.72)		674 (79)	179 (21)	

†Numbers may not sum to 100 due to rounding. ‡Education clustered into Lower (high school or less), Middle (up to 2 years at post secondary level) or Higher (over 2 years at post secondary level). NA (n=5) not included. §Country of birth with response options; Sweden, Europe or Outside Europe clustered into Sweden and Other. NA (n=8) not included. ¶Respondents' answers to the question; "Do you have children?". NA (n=5) not included. ††Self-reported personal cancer history with response alternatives yes (have been diagnosed with cancer) no (no current or previous cancer diagnosis), or "prefer not to say". NA (n=8) not included.

Table S8. Scores of the 8-item CWS in the population-based sample

		8-item CWS score			8-item CWS score interval		
	Subgroup	Median (min-max)	Mean (stand.dev.)	P-value t-test/ ANOVA	Low (8-item CWS score 6-13) N (%)†	High (8-item CWS score 14-32) N (%)†	P-value Chi2
**Total**	-	13 (8-32)	13.26 (3.85)		553 (59)	390 (41)	
**Gender**	Women	13 (8-30)	13.96 (4.14)	*P*<.001**	230 (51)	218 (49)	*P*<.001**
	Men	12 (8-32)	12.63 (3.46)		323 (65)	172 (35)	
	NA				0	0	
**Age**	18-29	12 (8-26)	12.80 (3.71)	*P*=.95	83 (67)	40 (33)	*P*=.47
	30-39	13 (8-30)	13.70 (4.38)		78 (55)	63 (45)	
	40-49	12 (8-32)	13.28 (4.20)		97 (61)	63 (39)	
	50-59	13 (8-27)	13.51 (3.76)		88 (57)	67 (43)	
	60-69	13 (8-27)	13.18 (3.63)		118 (58)	87 (42)	
	70-74	13 (8-25)	13.11 (3.47)		89 (56)	70 (44)	
	NA				0	0	
**Education**‡	Lower	13 (8-30)	13.26 (3.86)	*P*=.12	222 (59)	156 (41)	*P*=.95
	Middle	13 (8-32)	13.56 (4.00)		162 (54)	139 (46)	
	Higher	12 (8-24)	12.90 (3.62)		166 (64)	93 (36)	
	NA				3	2	
**Country of birth**§	Sweden	13 (8-32)	13.22 (3.80)	*P*=.40	512 (59)	360 (41)	*P*=.99
	Other	12 (8-27)	13.71 (4.56)		37 (59)	26 (41)	
	NA				4	4	
**Children**¶	Yes	13 (8-32)	13.41 (3.80)	*P*=.07	352 (57)	268 (43)	*P*=.10
	No	12 (8-30)	12.93 (3.91)		199 (63)	119 (37)	
	NA				2	3	
**Cancer history**††	Yes	14 (8-32)	14.82 (4.55)	*P*=.0013**	34 (41)	48 (59)	*P*=.0015**
	No	13 (8-30)	13.09 (3.70)		514 (60)	339 (40)	
	NA				5	3	

†Numbers may not sum to 100 due to rounding. ‡Education clustered into Lower (high school or less), Middle (up to 2 years at post secondary level) or Higher (over 2 years at post secondary level). §Country of birth with response options; Sweden, Europe or Outside Europe clustered into Sweden and Other. ¶Respondents' answers to the question; "Do you have children?". ††Self-reported personal cancer history with response alternatives yes (have been diagnosed with cancer) no (no current or previous cancer diagnosis), or "prefer not to say".
