# Supplementary material for: Cancer Worry Distribution and Willingness to Undergo Colonoscopy at Three Levels of Hypothetical Cancer Risk—A Population-Based Survey in Sweden
Source: Cancers (Basel). 2022 Feb 12;14(4):918. doi: 10.3390/cancers14040918 (PMC8870195; doi:10.3390/cancers14040918)
Supplement: Supplementary file 1 [file cancers-14-00918-s001.zip › cancers-1575752-supplementary_file2_surveyinvitation.pdf]

**Appendix A.** Survey invitation, English translation and Swedish original below.

*(Translated version, from Swedish)*

## Invitation to the Citizen Panel 31

Hello,

This is the next step of the Citizen Panel from Gothenburg University. The survey contains a mix of questions on attitudes and behaviors in society and is conducted in collaborations with researchers. Your answers are equally important to us no matter of your personal level of interest in societal issues. Everyone who participates in the survey will not receive exactly the same questions, this is decided at random.

You can access the survey through this link:

To the Citizen Panel

If the link does not work you can copy the following address into the url-window of your browser:

https://samgu/eu/qualtrics.com/jfe/preview/SV_cUNRe25I8wag7R4?Q_CHL=preview

This survey will take approximately X minutes to answer. Results will be presented in the form of tables and graphs without any possibility to identify any individual person's response. Collected data will be save for at least 10 years in order to enable data audit. Analyzed results will eventually be published in articles, papers, books and dissertations from Gothenburg University and collaborating research groups.

We look forward to your responses!

Johan Martinsson, Associate Professor
Head of Research for the Citizen Panel
Gothenburg University

---

*(Original version, in Swedish)*

## Inbjudan till Medborgarpanelen 31

Hej!

Här kommer nästa steg av Medborgarpanelen från Göteborgs universitet. Undersökningen innehåller en blandning av frågor kring attityder och beteende i samhället och görs i samarbete med forskare. Dina svar är lika viktiga för oss oavsett hur intresserad du är av samhällsfrågor. Alla som deltar i undersökningen får inte alltid exakt samma frågor, utan detta avgörs delvis av slumpen.

Du kommer till undersökningen via följande länk:
Till Medborgarpanelen

Om länken ovan inte fungerar kan du istället kopiera in följande adress i adressfältet på din webbläsare:
https://samgu.eu.qualtrics.com/jfe/preview/SV_cUNRe25I8wag7R4?Q_CHL=preview

Denna undersökning tar cirka  minuter att besvara. Resultaten presenteras i form av tabeller och figurer där det inte framgår vad någon enskild person har svarat. Datan som samlas in sparas i minst 10 år för att möjliggöra granskning. Analyser av resultaten kommer så småningom att publiceras i artiklar, uppsatser, böcker och avhandlingar från Göteborgs universitet och samverkande forskningsgrupper.

Vi ser fram emot dina svar!

Med vänliga hälsningar,

Johan Martinsson, docent
Forskningsledare för Medborgarpanelen
Göteborgs universitet

Om du har frågor kan du nå oss via e-post info@medborgarpanelen.gu.se eller besöka vår hemsida www.medborgarpanelen.gu.se

Om du vill ändra e-postadress och framöver få våra utskick till en annan mejladress kan du göra det på följande adress: www.medborgarpanelen.gu.se/epost

Känner du någon mer som du tror är intresserad av att delta i undersökningar liknande denna? De kan i så fall enkelt anmäla sig till Göteborgs universitets Medborgarpanel via följande länk: www.medborgarpanelen.gu.se/anmalan

Om du inte vill delta i fler undersökningar från Medborgarpanelen kan du avregistrera dig via följande länk: www.medborgarpanelen.gu.se/avregistrering
